# Comparative Evaluation of Bruker Biotyper and ASTA MicroIDSys for Species Identification in a Clinical Microbiology Laboratory

**DOI:** 10.3390/diagnostics11091683

**Published:** 2021-09-15

**Authors:** Yousun Chung, Minje Han, Jae-Seok Kim

**Affiliations:** 1 Department of Laboratory Medicine, Kangdong Sacred Heart Hospital, Seoul 05355, Korea; yousun623@kdh.or.kr (Y.C.); mjhan@kdh.or.kr (M.H.); 2 Department of Laboratory Medicine, Hallym University College of Medicine, Chuncheon 24252, Korea

**Keywords:** matrix-assisted laser desorption/ionization time-of-flight mass spectrometry (MALDI–TOF MS), microorganism, identification

## Abstract

Matrix-assisted laser desorption/ionization time-of-flight mass spectrometry (MALDI–TOF MS) has been widely used for microbial identification, because of its speed and accuracy, since its introduction to clinical microbiology laboratories. In this study, we evaluated the performance of ASTA MicroIDSys, a newly developed MALDI–TOF, and compared it with the widely used Bruker Biotyper. Microbial identification with the Bruker Biotyper system was performed by using a direct smear method and the Bruker Biotyper database (reference library version 6.0.0.0). The isolates were also tested in parallel, using the ASTA MicroIDSys system with a direct smear method and the MicroIDSys database, CoreDB v1.26. A total of 914 clinical isolates were recovered from the clinical specimens. Identical results with confidence scores (≥2.0, for the Bruker Biotyper) and acceptable scores (≥140 for the ASTA MicroIDSys) were obtained for 840 (91.9%) isolates. The minor errors were defined as misidentification at the species level, and the rate was 1.1% (9/792) for Bruker Biotyper and 1.6% (13/792) for ASTA MicroIDSys. Major errors were defined as misidentification at the genus level, and the rate was 0.3% (2/792) for both Bruker Biotyper and ASTA MicroIDSys. ASTA MicroIDSys showed reliable performance for microbial identification, which was comparable to that of the Bruker Biotyper. Therefore, ASTA MicroIDSys can be applied for the identification of microorganisms in clinical microbiology laboratories.

## 1. Introduction

The introduction of matrix-assisted laser desorption/ionization time-of-flight mass spectrometry (MALDI–TOF MS) has greatly improved the turnaround time for the routine identification of microorganisms, along with rapid and accurate identification [1,2,3]. MALDI–TOF MS provided consistent and accurate results when compared with those of biochemical identification methods, but no standard method for dealing with unexpected species identification has been presented. The discrepancies in the outcomes for rare bacterial species for different MALDI–TOF MS manufacturers should be examined.

There are two commercially available MALDI–TOF MS systems: the Microflex Biotyper (Bruker Daltonics, Bremen, Germany) and VITEK MS (bioMérieux, Marcy l’Etoile, France), which are implemented in clinical microbiology laboratories worldwide [4,5,6,7]. Recently, a new MALDI–TOF MS system, ASTA MicroIDSys (ASTA Inc., Suwon, Korea), was developed for the identification of clinically important microorganisms.

In the present study, we compared the routine performance of the ASTA MicroIDSys with that of the Microflex Biotyper for identification of all microbial isolates, including bacteria and yeasts, in a clinical microbiology laboratory, during the study period.

## 2. Materials and Methods

All clinical strains, except for filamentous fungi and mycobacteria, isolated in a clinical microbiology laboratory at a 750-bed general hospital in Korea, from October to December 2018, were included in this study. The study was approved by the institutional review board of Kangdong Sacred Heart Hospital (IRB file No. Kangdong NON2018-001, 18 September 2018). The clinical specimens were inoculated in appropriate media, such as 5% sheep blood agar, MacConkey agar, or chocolate agar for bacteria; Buccella blood agar for anaerobic bacteria; and Sabouraud dextrose agar for yeast. The specimens were then incubated for 24–48 h, at 35 °C, in appropriate conditions. A total of 914 clinical isolates were recovered from clinical specimens of blood, body fluids, wounds, and pus.

Microbial identification with Bruker Biotyper system was performed by using a direct smear method, following the manufacturer’s instructions. Briefly, a singly colony of the isolate was smeared and dried on a plate. Subsequently, 70% formic acid and cyano-4-hydroxycinnamic acid matrix solution were added, and the target plate was analyzed by using Bruker Biotyper database (reference library version 6.0.0.0, Bruker Daltonics, Bremen, Germany). The confidence scores values over 2.0 were considered acceptable according to the manufacturer’s recommendations. The isolates were also tested in parallel, using ASTA MicroIDSys system with a direct smear method. Moreover, the target plate was analyzed by using MicroIDSys database (CoreDB v1.26, ASTA Inc., Suwon, Korea). Identification scores over 140 were considered acceptable according to the manufacturer’s recommendations. If the test provided a score under the target cutoff (<140 for MicroIDSys and <2.0 for Bruker Biotyper) or invalid results, we immediately repeated the test with other colonies from the same agar plate.

When the results by two systems showed discrepancies at the species level or one of the results was under the cutoff score or invalid, 16S rRNA gene sequencing was performed for bacterial identification by Macrogen (Seoul, Korea). The PCR primers for 16S rRNA were 5′-GGATTAGATACCCTGGTA-3′ and 5′-CCGTCAATTCMTTTRAGTTT-3′, and the sequencing primers were 5′-AGAGTTTGATCMTGGCTCAG-3′ and 5′-TACGGYTACCTT GTTACGACTT-3′. The 16S rRNA sequences obtained were compared with GenBank data, using the BLAST alignment software (blast.ncbi.nlm.nih.gov, accessed on 12 July 2021) and a threshold of ≥99% homology was used for identification to the species level.

Chi-square test or Fisher’s exact test was used for analyzing differences in the identification rate. SPSS Statistics 24 (IBM SPSS Inc., Chicago, IL, USA) was used for statistical analyses, and a 2-tailed *p*-value of 0.05 was considered statistically significant.

## 3. Results

The isolates were identified as belonging to Gram-negative bacilli (*N* = 417, 45.6%), Gram-positive cocci (*N* = 329, 36.0%), and other bacteria (*N* = 60, 6.6%), and fungi (*N* = 108, 11.8%). The most frequently isolated bacteria were *Escherichia coli* (*N* = 136, 14.9%), followed by *Staphylococcus aureus* (*N* = 99, 10.8%), *Klebsiella pneumoniae* (*N* = 79, 8.6%), *Enterococcus faecium* (*N* = 63, 6.9%), *Acinetobacter baumannii* (*N* = 48, 5.3%), *Pseudomonas aeruginosa* (*N* = 46, 5.0%), *Candida tropicalis* (*N* = 42, 4.6%), *Candida albicans* (*N* = 36, 3.9%), *E. faecalis* (*N* = 33, 3.6%), *S. epidermidis* (*N* = 25, 2.7%), *Corynebacterium striatum* (*N* = 25, 2.7%), *Candida glabrata* (*N* = 19, 2.1%), *S. haemolyticus* (*N* = 18, 2.0%), *Enterobacter aerogenes* (*N* = 15, 1.6%), and *Streptococcus anginosus* (*N* = 14, 1.5%).

From the 914 isolates analyzed, identical results with confidence scores (≥2.0 for the Bruker Biotyper) and acceptable scores (≥140 for the ASTA MicroIDSys) were obtained for 840 (91.9%) isolates. After applying lower confidence scores (≥1.7) for the Bruker Biotyper, 24 (94.5%) additional isolates showed identical results for the two systems (Table 1).

The correct identification rate at the species level was evaluated for the results of 792 bacterial isolates, as 16S rRNA sequencing could not be performed for 108 fungal isolates and 14 bacterial isolates because of the lack of samples. The correct identification rate with a score above the target cutoff (≥2.0, Bruker Biotyper, and ≥140 for MicroIDSys and) was 94.2% (746/792) by Bruker Biotyper and 95.7% (758/792) by ASTA MicroIDSys (*p* = 0.177).

Minor errors were defined as misidentification at the species level with a score above the target cutoff (≥2.0 for Bruker Biotyper and ≥ 140 for MicroIDSys). The minor error rate was 1.1% (9/792) for Bruker Biotyper and 1.6% (13/792) for ASTA MicroIDSys (*p* = 0.388). Major errors were defined as misidentification at the genus level (≥140 for MicroIDSys and ≥ 1.7 for Bruker Biotyper). Exceptionally, the misidentification of *S. aureus* at the species level was considered as a major error because of the clinical importance of this species. The major error rate was 0.3% (2/792) for both Bruker Biotyper and ASTA MicroIDSys, and there was no case of misidentification of *S. aureus* as other coagulase-negative staphylococci. The isolate of *Weisella cibaria* was misidentified as *E. coli*, and the isolate of *Brevibacterium frigoritolerans* as *Lactobacillus jensenii* by Bruker Biotyper. The isolate of *Staphylococcus warneri* was misidentified as *Azotobacter nigricans* and the isolate of *Kluyvera ascorbata* was misidentified as *Raoultella ornithinolytica* by ASTA MicroIDSys.

The two systems showed discrepant results for 31 isolates (3.4%). However, at the genus level, they were in agreement, except for five isolates. The identification results for the 16S rRNA gene sequencing for these isolates are shown in Table 2. The 16S rRNA sequencing could not be performed for 14 bacterial isolates due to the lack of samples and one fungal isolate. Among the 16 results for the 16S rRNA sequencing, nine and three were in agreement with those of Bruker Biotyper and ASTA MicroIDSys, respectively (*p* = 0.066).

The Bruker Biotyper showed invalid results for 10 isolates (1.1%), and ASTA MicroIDSys showed invalid results for four isolates (0.4%) (*p* = 0.178). The identification results for the 16S rRNA gene sequencing for these isolates are shown in Table 3.

## 4. Discussion

In this study, we evaluated the performance of ASTA MicroIDSys, a newly developed MALDI–TOF system which can be routinely used in clinical microbiology laboratories, in comparison with Bruker Biotyper which is widely used. Most of the bacteria and yeasts which are commonly isolated in clinical laboratories were correctly identified by MALDI–TOF MS, while a few uncommon bacteria, including *Brevibacterium* spp., *Pseudoglutamicibacter* spp., and *Janibacter* spp., were given invalid results.

The performance of MicroIDSys was comparable to that of Bruker Biotyper with the overall concordance rate of 91.9%. This result is in the same line with a previous study which reported good agreement of results between Bruker Biotyper and ASTA MicroIDSys. In that study, identical results with confidence scores (≥2.0 for Bruker Biotyper) and acceptable scores (≥140 for ASTA MicroIDSys) were obtained for 86.1% from the 4919 isolates recovered from sputum, urine, and pus samples [8]. In another study which evaluated the performance of ASTA MicroIDSys compared to that of VITEK MS, the ASTA MicroIDSys correctly identified 96.7% of isolates to species level which was comparable to VITEK MS (97.3%) [9].

As microorganisms are identified by MALDI–TOF MS systems, using prerecorded protein spectra, which are mostly based on ribosomal proteins, MALDI–TOF MS systems are intrinsically limited to differentiate closely related species of *Klebsiella*, *Enterobacter*, *Citrobacter*, and *Raoultella* [10,11]. The discrepant results between Bruker Biotyper and ASTA MicroIDSys in this study also revealed the known limitation of MALDI–TOF systems. Successful identification of microorganisms by using MALDI–TOF MS relies heavily on the database containing the spectra of known organisms. It is critical that it includes a sufficient number of isolates for each species, grown under a variety of conditions such that the spectral library for the organism is sufficiently robust to account for the inherent variability expected for any organism. Most of the discrepant or invalid results in this study were from microorganisms that are not frequently isolated in clinical laboratories. Of note, the ASTA MicroIDSys misidentified the rare microorganisms with scores over 140. There should be caution when interpreting the results of ASTA MicroIDSys with microorganisms such as *Paenibacillus* spp. or *Weissella* spp.

Molecular approaches could be useful for correct identification. There has been a study which applied whole genome-based bacterial identification system for clinical isolates that were not identified with MALDI–TOF MS systems [12]. It evaluated thirty-six isolates including *Corynebacterium* spp., *Brevibacterium* spp., and *Brevundimonas* spp. which were also not correctly identified by MALDI–TOF MS in our study. Genome-based identification may be an additional tool in the future. However, whole genome sequencing is yet burdensome in cost and methodology for clinical microbiology laboratories. Targeted sequencing of 16S rRNA, *gyrB*, or *rpoB* for bacteria, and internal transcribed spacer (ITS) or 28S region for yeasts can be the practical approach when MALDI–TOF MS cannot give the correct identification.

Except for the above mentioned studies, there are other studies that evaluated the performance of ASTA MicroIDSys on yeast [13], anaerobic bacteria [14], mycobacteria [15], and filamentous fungi [16]. As the aim of this study was to evaluate the utilization of ASTA MicroIDSys for routine identification in clinical microbiology laboratories, there was no selection on the types of microorganisms or on the types of samples. This study evaluated the performance of ASTA MicroIDSys by using microorganisms isolated from all types of samples including blood specimen at a 750-bed general hospital.

There are several limitations in this study. First, when the results obtained with the Bruker Biotyper and the ASTA MicroIDSys were identical at the species with scores above the target cutoff, we considered the results as correct identification without performing16S rRNA sequencing. Second, there is inborn limitation of 16S rRNA sequencing as it may show poor discrimination power for some genera in Gram-positive cocci [17], *Enterobacteriaceae* [18], or for *Campylobacter* spp. [19]. However, the BLAST alignment of 16S rRNA sequencing showed only one type of species with ≥99% homology for all the clinical isolates in this study. Third, 16S rRNA sequencing could not be performed for 14 bacterial isolates which showed discrepant results by the Bruker Biotyper and ASTA MicroIDSys due to lack of samples. Lastly, molecular testing for fungal isolates could not be performed due to our laboratory setting, which might limit the exact evaluation of performance for identification of fungi. However, as 100 (95.2%) among the 105 isolates of *Candida* spp. showed concordant results at the species level by the Bruker Biotyper and the ASTA MicroIDSys with scores above the target cutoff, it was demonstrated that ASTA MicroIDSys could reliably identify clinically important fungal isolates.

## 5. Conclusions

Since the introduction of MALDI–TOF MS in clinical microbiology laboratories, it has been widely used for microbial identification because of its speed and accuracy. In this study, ASTA MicroIDSys showed reliable performance on microbial identification that was comparable to that of the Bruker Biotyper. Therefore, ASTA MicroIDSys could be applied for identification of microorganism in clinical microbiology laboratories.

## Figures and Tables

**Table 1:** Comparison of the results of the Bruker Biotyper and ASTA MicroIDSys systems.

Species	Bruker ≥ 2.0		Bruker 1.7≤, <2.0		Total
	ASTA ≥ 140	ASTA < 140	ASTA ≥ 140	ASTA < 140	
Gram-negative bacilli					
*Escherichia coli*	134	1		1 * ^1^	136
*Klebsiella pneumoniae*	79				79
*Acinetobacter baumannii*	48				48
*Pseudomonas aeruginosa*	45		1		46
*Proteus mirabilis*	18				18
*Enterobacter aerogenes*	15				15
*Enterobacter cloacae*	9				9
*Serratia marcescens*	8				8
*Stenotrophomonas maltophilia*	7				7
*Haemophilus influenzae*	5				5
*Citrobacter freundii*	4				4
*Acinetobacter baylyi*			3 * ^2^		3
*Providencia rettgeri*	2				2
*Alcaligenes faecalis*	2				2
*Morganella morganii*	2				2
*Citrobacter amalonaticus*	2				2
*Achromobacter xylosoxidans*	2				2
*Acinetobacter nosocomialis*	2				2
*Acinetobacter pittii*	1		1 * ^3^		2
*Providencia stuartii*	1				1
*Salmonella spp.*	1				1
*Aeromonas veronii*	1				1
*Pseudomonas stutzeri*	1				1
*Neisseria gonorrhoeae*	1				1
*Aeromonas caviae*	1				1
*Campylobacter jejuni*	1				1
*Haemophilus parainfluenzae*	1				1
*Brevundimonas vesicularis*			1 * ^4^		1
No. of subtotal (%)	393 (98.0%)	1 (0.2%)	6 (1.5%)	1 (0.2%)	401 (100%)
Gram-positive cocci					
*Staphylococcus aureus*	99				99
*Enterococcus faecium*	63				63
*Enterococcus faecalis*	33				33
*Staphylococcus epidermidis*	25				25
*Staphylococcus haemolyticus*	14		4		18
*Streptococcus anginosus*	10		4		14
*Streptococcus agalactiae*	10				10
*Staphylococcus hominis*	8		1		9
*Staphylococcus lugdunensis*	7				7
*Staphylococcus capitis*	7				7
*Staphylococcus caprae*	5		2		7
*Enterococcus casseliflavus*	4				4
*Staphylococcus pettenkoferi*	3				3
*Streptococcus pyogenes*	2				2
*Enterococcus avium*	2				2
*Streptococcus constellatus*	2				2
*Enterococcus raffinosus*	2				2
*Streptococcus mitis*	2				2
*Streptococcus salivarius*	1		1		2
*Streptococcus pneumoniae*	1	1			2
*Staphylococcus simulans*	1				1
*Micrococcus luteus*	1				1
*Streptococcus dysgalactiae*	1				1
*Enterococcus gallinarum*	1				1
*Streptococcus intermedius*	1				1
*Streptococcus parasanguinis*	1				1
No. of subtotal (%)	306 (95.9%)	1 (0.3%)	12 (3.8%)	0 (0.0%)	319 (100%)
Other bacteria					
*Corynebacterium striatum*	25				25
*Clostridium difficile*	9		1		10
*Corynebacterium jeikeium*	1			1	2
*Clostridium hathewayi*	1				1
*Bacillus cereus*	1				1
*Actinomyces odontolyticus*	1				1
*Bacillus circulans*	1				1
*Corynebacterium tuberculostearicum*			1		1
No. of subtotal (%)	39 (92.9%)	0 (0.0%)	2 (4.8%)	1 (2.4%)	42 (100%)
*Candida* spp. and other fungi ^†^					
*Candida tropicalis*	41		1		42
*Candida albicans*	35		1		36
*Candida glabrata*	18		1		19
*Candida parapsilosis*	6		1		7
*Trichosporon asahii*	2				2
*Cryptococcus neoformans*				1	1
No. of subtotal (%)	102 (95.3%)	0 (0.0%)	4 (3.7%)	1 (0.9%)	107 (100%)
No. of total (%)	840 (96.7%)	2 (0.2%)	24 (2.8%)	3 (0.3%)	869 (100%)

* When any result was below the cutoff score (<140 for MicroIDSys and <2.0 for Bruker Biotyper), we performed 16S rRNA gene sequencing for bacterial identification. The results of the *16S rRNA* gene sequencing are provided in parentheses for the cases whose results by molecular testing were different from those by MALDI–TOF MS (^1^ *E. fergusonii*; ^2^ *A. soli*; ^3^ *A. calcoaceticus*; ^4^ *B. nasdae*). ^†^ Molecular testing was not performed for fungal isolates.

**Table 2:** List of isolates with discrepant results by the Bruker Biotyper and ASTA MicroIDSys systems.

	Bruker		ASTA		Identification by 16S rRNA Sequencing (Accession)
	Identification	Score	Identification	Score	
Concordant at genus level	*Klebsiella variicola*	1.984	*Klebsiella pneumoniae*	203	*Klebsiella variicola* (CP010523.2)
	*Klebsiella variicola*	2.111	*Klebsiella pneumoniae*	177	*Klebsiella variicola* (CP010523.2)
	*Klebsiella variicola*	2.264	*Klebsiella pneumoniae*	236	N/T
	*Klebsiella variicola*	2.242	*Klebsiella pneumoniae*	144	N/T
	*Klebsiella variicola*	1.903	*Klebsiella pneumoniae*	157	N/T
	*Streptococcus pneumoniae*	2.216	*Streptococcus mitis*	194	*Streptococcus pneumoniae* (LN831051.1)
	*Streptococcus pneumoniae*	2.117	*Streptococcus mitis*	176	*Streptococcuspneumoniae* (NR_028665.1)
	*Streptococcus pneumoniae*	2.144	*Streptococcus mitis*	169	*Streptococcus mitis* (NR_028665.1)
	*Streptococcus pneumoniae*	1.894	*Streptococcus sobrinus*	111	*Streptococcus mitis* (NR_028664.1)
	*Streptococcus vestibularis*	2.149	*Streptococcus salivarius*	223	*Streptococcus salivarius* (CP009913.1)
	*Streptococcus infantis*	1.884	*Streptococcus mitis*	169	*Streptococcus infantis* (LC096227.1)
	*Streptococcus oralis*	2.056	*Streptococcus mitis*	160	N/T
	*Enterobacter asburiae*	2.111	*Enterobacter cloacae*	207	*Enterobacter kobei* (CP017181.1)
	*Enterobacter asburiae*	2.151	*Enterobacter cloacae*	206	N/T
	*Enterobacter kobei*	2.263	*Enterobacter cloacae*	207	N/T
	*Citrobacter youngae*	2.108	*Citrobacter freundii*	179	*Citrobacter braakii* (NR_028687.1)
	*Citrobacter koseri*	2.291	*Citrobacter amalonaticus*	226	N/T
	*Paenibacillus urinalis*	2.127	*Paenibacillus macerans*	112	*Paenibacillus urinalis* (NR_044178.1)
	*Paenibacillus urinalis*	2.247	*Paenibacillus lactis*	153	*Paenibacillus urinalis* (NR_044178.1)
	*Paenibacillus barengoltzii*	2.167	*Paenibacillus macerans*	171	*Paenibacillus barengoltzii* (NR_113988.1)
	*Paenibacillus glucanolyticus*	1.931	*Paenibacillus ginsengagri*	196	N/T
	*Pseudomonas monteilii*	2.084	*Pseudomonas putida*	181	N/T
	*Burkholderia lata*	2.155	*Burkholderia cepacia*	198	N/T
	*Corynebacterium simulans*	2.237	*Corynebacterium striatum*	151	N/T
	*Providencia rettgeri*	1.833	*Providencia stuartii*	168	N/T
	*Candida metapsilosis*	1.708	*Candida orthopsilosis*	134	N/T
Discordant at the genus level	*Kluyvera ascorbata*	2.128	*Raoultella ornithinolytica*	167	*Kluyvera ascorbata* (NR_028677.1)
	*Escherichia coli*	2.059	*Weissella confusa*	239	*Weissella cibaria* (LC096236.1)
	*Staphylococcus warneri*	1.970	*Azotobacter nigricans*	143	*Staphylococcus warneri* (NR_025922.1)
	*Streptococcus pneumoniae*	2.073	*Saccharomyces cerevisiae*	120	N/T
	*Clostridium difficile*	1.961	*Eggerthella lenta*	171	N/T

**Table 3:** List of isolates whose identification showed invalid results by the Bruker Biotyper or ASTA MicroIDSys systems.

	Bruker		ASTA		Identification by 16S rRNA Sequencing (Accession)
	Identification	Score	Identification	Score	
Correct identification by Bruker *	*Pantoea calida*	2.236	Invalid Identification		*Pantoea calida* (AB907785.1)
	*Weeksella virosa*	2.184	Invalid Identification		*Weeksella virosa* (CP002455.1)
	*Streptococcus mitis*	1.805	Invalid Identification		*Streptococcus mitis* (NR_028664.1)
Correct identification by ASTA *	Invalid Identification		*Propionibacterium acnes*	200	*Propionibacterium acnes* (CP003084.1)
	Invalid Identification		*Moraxella osloensis*	160	*Moraxella osloensis* (CP014234.1)
	Invalid Identification		*Weissella confusa*	203	*Weissela cibaria* (LC096236.1)
	Invalid Identification		*Brevibacillus centrosporus*	134	*Brevibacillus limnophilus* (NR_024822.1)
	Invalid Identification		*Paenibacillus lactis*	115	*Paenibacillus* spp. (JN377815.1)
Incorrect identification	*Lactobacillus jensenii*	2.003	Invalid Identification		*Brevibacterium frigoritolerans* (NR_117474.1)
	Invalid Identification		*Staphylococcus arlettae*	117	*Pseudoglutamicibacter cumminsii* (NR_044895.1)
	Invalid Identification		*Parvimonas micra*	125	*Dermabacter vaginalis* (CP012117.1)
	Invalid Identification		*Bacillus simplex*	131	*Brevibacterium frigoritolerans* (NR_117474.1)
	Invalid Identification		*Knoellia subterranea*	132	*Janibacter hoylei* (NR_104794.1)
	Invalid Identification		*Paenibacillus timonensis*	125	*Lysinibacillus* spp. (HE586367.1)

* It was regarded as correct identification when the results by Bruker or ASTA was concordant with the results by 16S rRNA sequencing at the genus level.

## Data Availability

Data is contained within the article.
